# Enhanced hierarchical attention networks for predictive interactome analysis of LncRNA and CircRNA in oral herpes virus

**DOI:** 10.1016/j.jobcr.2025.02.012

**Published:** 2025-03-10

**Authors:** Pradeep Kumar Yadalam, Carlos M. Ardila

**Affiliations:** a Department of Periodontics, Saveetha Dental College, Saveetha Institute of Medical and technology sciences, SIMATS, Saveetha. University, Chennai, Tamil Nadu, India; b Department of Basic Sciences, Biomedical Stomatology Research Group, Faculty of Dentistry, Universidad de Antioquia U de A, Medellín, Colombia

**Keywords:** Long coding RNA, Cir-RNA, Herpes virus, Graph attention networks

## Abstract

**Background:**

Non-coding RNAs, including lncRNAs, circRNAs, and microRNAs, constitute 98 % of the human transcriptome and are vital regulators of gene expression, cellular processes, and host-pathogen interactions, particularly in viral infections. This study explores lncRNA-circRNA interactions and their biological significance in oral viral infections.

**Methods:**

ViRBase, a database with over 820,000 interactions involving 50,000 RNAs from 116 viruses and 36 host organisms, was used to analyze herpesvirus datasets. The study employed hierarchical attention and knowledge graph embeddings to represent nodes and edges in the knowledge graph. These served as input features for a hierarchical attention model trained over 100 epochs. Model performance was evaluated based on loss calculation, optimization, and attention weight stability.

**Results:**

The model achieved a final loss of 0.000180 at Epoch 100, with stable attention weights confirming reliability. Node embedding statistics showed a mean of 0.005110 and a standard deviation of 0.013370, while attention weights had a high mean of 0.997178, emphasizing model robustness.

**Conclusion:**

This study provides insights into lncRNA-circRNA interactions in herpes viral infections, enhancing therapeutic development, disease progression monitoring, and understanding host-pathogen interactions, paving the way for targeted interventions and improved outcomes.

## Graphical abstract

**Figure:**
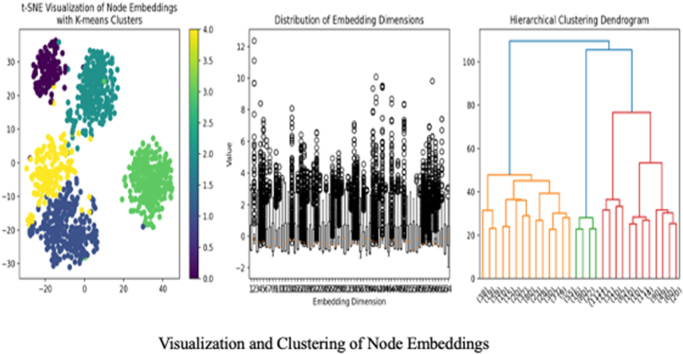

## 1 Introduction

Herpesviruses are large, enveloped, double-stranded DNA viruses that can cause lifelong infections. They belong to three families: alpha, beta, and gamma. These viruses alternate between lytic and latent phases to evade the host's immune system, aided by viral non-coding RNAs (ncRNAs).^1^ These ncRNAs optimize the herpesvirus's limited genetic material, serving multifunctional roles while being largely non-immunogenic, which allows their expression even when host protein translation is halted. This manipulation of gene expression is vital for the herpesvirus life cycle, and research is ongoing to understand the various roles of ncRNAs in viral replication. Their roles in viral infections, particularly in modulating host immune responses and facilitating viral replication, have garnered significant attention.^2^, ^3^, ^4^, ^5^ CircRNAs (circular RNAs) play significant roles in various biological processes, including acting as sponges for microRNAs and RNA-binding proteins, mediating alternative splicing, regulating parental gene expression, and even translating small peptides. During virus-host interactions, various antiviral mechanisms have evolved in host immune systems, such as RNA interference and interferon pathways, to combat viral infections. A recent novel antiviral mechanism involves circRNA recognition by the RIG-I receptor, activating immune responses and potentially suppressing viral replication, revealing similarities between host-derived and viral circRNAs in regulatory functions.^6^^,^^7^

Non-coding RNAs (ncRNAs) are transcribed from DNA but are not translated into proteins, with two primary categories based on length: short non-coding RNAs (less than 200 nucleotides) and long non-coding RNAs (lncRNAs, more than 200 nucleotides). A new deep learning framework, circDeep,^8^ has been introduced to improve classification accuracy and processing times, achieving a 12 % increase in classification accuracy and faster processing times. Long non-coding RNAs (lncRNAs) and microRNAs interact in complex ways. Deep learning methods like CIRNN and PmliPred improve LMI prediction, while graph-based approaches like GCLMI and GEEL-FI use heterogeneous graphs for interaction prediction. The LncMirNet method combines feature extraction and CNN for improved bioinformatics LMI prediction. One previous study introduces KGDCMI, a new method for predicting circRNA-miRNA interactions^9^ using multi-source data. It uses a graph-embedding algorithm, principal component analysis, and a deep neural network. The method outperforms existing models by 2.37 % and 3.08 % and validates 7 of the top 10 predicted interaction pairs, making it valuable for RNA biological research.^6^

ViRBase,^10^ a database examining non-coding RNA interactions in viral infections, has version 3.0 and catalogs over 820,000 interactions between 116 viruses and 36 host organisms. The database includes information on unique identifiers, virus and strain names, taxonomy IDs, virus families, and host species. It also provides references and confidence scores for each entry, aiding in understanding viral interactions.ViRBase v3.0 has expanded its database to over 820,000 interaction entries, a 70-fold increase from its previous version. The update includes RNA annotations, ncRNA single nucleotide polymorphism (SNP) and drug-related information, two new tools for predicting binding sites, a visual plug-in for interaction display, and a user-friendly website.

Non-coding RNAs (lncRNAs) and circular RNAs (circRNAs) are crucial regulators in gene expression, cellular processes, and host-pathogen interactions. Long non-coding RNAs (lncRNAs) play critical roles in herpesvirus infections, particularly in Human Cytomegalovirus (HCMV) and Herpes Simplex Virus (HSV).^7^^,^^8^ They assist in viral replication, evade host defenses, and maintain latency. HCMV RNA2.7 interacts with mitochondrial Complex I to support ATP production under stress, while HSV's lncRNA LAT prevents apoptosis in infected neurons. Additionally, lncRNAs like HCMV RNA4.9 and KSHV PAN affect chromatin remodeling to regulate viral gene expression. These lncRNAs are essential for both latency maintenance and the regulation of viral genes during infection. This study investigates the interactions between lncRNAs, circRNAs, and their associated RNAs, focusing on their biological significance in oral herpes virus.^6^^,^^7^ Predicting RNA interactions is essential, and these interactions can be sequence-based or network-based. Sequence-based methods analyze RNA nucleotide sequences to identify binding sites through motif finding, base pairing predictions, and sequence alignment.^6^^,^^8^ Network-based methods utilize existing interaction data, employing guilt-by-association, co-expression analysis, and pathway analysis to infer new interactions. Machine learning-based methods leverage algorithms that utilize feature engineering, supervised learning on labeled datasets, and deep learning techniques to uncover complex patterns in RNA sequences. Each method offers unique strategies for enhancing our understanding of RNA interactions in biology systems. Understanding these molecular mechanisms is crucial for developing effective therapeutic strategies and identifying potential biomarkers and therapeutic targets. The findings can provide novel insights into gene regulation, cellular processes, and host-pathogen interactions, potentially identifying new regulatory pathways and molecular mechanisms.

Hierarchical Graph Attention Networks (H-GATs) excel in modeling RNA interactions by capturing hierarchical relationships and aggregating information across multiple layers. Their attention mechanisms enhance interpretability by highlighting the significance of different interactions, aiding biologists in identifying key components in RNA signaling pathways. Additionally, H-GATs are computationally efficient, enabling quicker training and better resource use in large RNA datasets. Overall, they uniquely combine structure and clarity for RNA interaction modeling. H-GATs^11^, ^12^, ^13^ are a powerful tool for predictive interactome analysis, outperforming traditional AI methods. They capture hierarchical structures and intricate relationships within network data, enabling a more nuanced understanding of the network's dynamics. H-GATs also handle biological networks' complex, multi-scale nature, allowing the model to learn local and global patterns simultaneously. One previous study showed that the HGANMDA^14^ uses a hierarchical graph attention network to predict miRNA-disease associations. It uses node-layer and semantic-layer attention to evaluate neighboring nodes and meta-paths. A bilinear decoder reconstructs connections between miRNAs and diseases, outperforming existing methods in extensive experiments.

Their interpretability allows insights into underlying biological processes and key regulatory elements, particularly in lncRNA and circRNA research. The study also identifies key patterns and interactions within the interactome, which can inform the discovery of novel biomarkers for oral viral infections and potential therapeutic targets. This study aims to analyze the interactions between lncRNAs, circRNAs, and their associated RNAs, focusing on their biological significance in oral viral infections.

## 2 Materials and methods

### 2.1 Dataset retrieval

ViRBase^10^ is a resource that highlights the significant roles of non-coding RNAs (ncRNAs) in viral infections by documenting the interactions between viral and cellular ncRNAs. The current version, ViRBase v3.0, includes over 820,000 documented interactions, supported by experimental and predicted evidence, involving more than 50,000 RNAs from 116 viruses and 36 host organisms, primarily from families such as Flaviviridae, Polyomaviridae, Herpesviridae, Retroviridae, and Coronaviridae. This database aims to enhance the understanding of viral infections and aid in developing novel antiviral therapies. The selection of these interactions from the viral dataset is comprehensive and beneficial for researchers conducting in-depth analyses of coding and non-coding RNAs related to the herpes virus.

### 2.2 Merging and early fusion architecture

Downloaded data from two files, lncRNA_RNA_interactions and circRNA_RNA_interactions, were read into separate panda's data frames, with a new column named 'Source' added to each. The data was then concatenated into a single data frame,'merged_RNA_interactions.txt'. After updating the data frame with early fusion, saves it, creates additional fused features, creates numerical features, creates a feature matrix with all fused features, displays a summary of the fused features and generates a feature matrix, displays the first few rows, and saves the feature matrix as a CSV file. Combining raw features into a unified representation enhances feature interaction and understanding, simplifies pipeline management, and is more computationally efficient than late fusion, as it allows the model to work with a single input stream. Early fusion combines relevant features into a unified representation, creating a fused feature by concatenating the 'Interactor1 Symbol' and 'Interactor2 Symbol' columns. The fused features are then compiled into a feature matrix, which represents the interactions post-fusion and is ready for further analysis. Early fusion was performed by creating new features that combined existing columns. For example, the 'Interactor1 Symbol' and 'Interactor2 Symbol' columns were concatenated to form 'Fused_Feature'. Other fused features were created by combining the 'Virus Name' and 'Host Species' columns into 'Virus_Host_Feature,'' Interactor1 Category', and 'Interactor2 Category' columns into 'Category_Feature.' A numerical feature named 'Taxonomy_Score' was generated by multiplying 'Taxonomy ID' by 'Score.' Herpes viruses were filtered from the merged data, with results saved in separate files. No papillomavirus interactions were found, while herpesvirus yielded 3360 interactions. The selected herpesvirus types were chosen for their clinical and biological significance in oral and periodontal diseases, capturing variations in RNA interaction patterns and providing insights into herpesviruses' regulatory mechanisms.

**Fig. 1:**
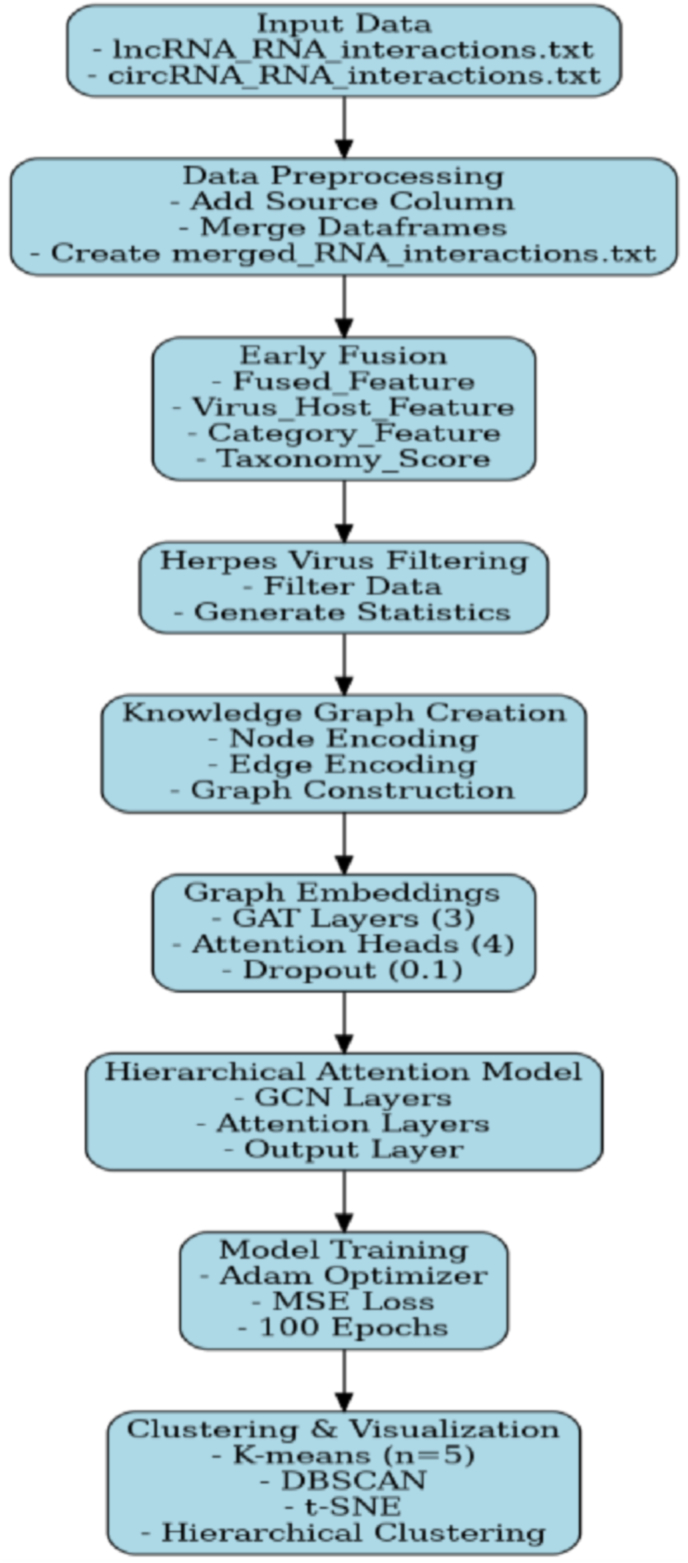
Workflow Architecture for Analyzing lncRNA-circRNA Interactions in Herpes Viral Infections.

Fig. 1 illustrates the workflow for analyzing lncRNA-circRNA interactions using hierarchical attention and knowledge graph embeddings. The process begins with input data consisting of RNA interaction datasets, followed by preprocessing to merge and create a unified interaction file.

### 2.3 Early fusion – graph embeddings with GAT

Early fusion integrates multiple features, including virus-host interactions and taxonomy scores. Herpesvirus-specific data are filtered for generating statistics. Developing a knowledge graph requires establishing nodes and edges, featuring virus nodes for herpesviruses, host species nodes for different host species, and interaction nodes that indicate specific relationships between viruses and hosts. These nodes supply information on virus names, taxonomic classification, and biological functions; in the herpesvirus dataset, edges can represent virus-host interactions, taxonomic relationships, and functional relationships. The knowledge graph is created using a graph database or library, initializing nodes with features, associating edges with weights and types, and structuring for efficient traversal and analysis. The hierarchical attention model, incorporating GCN layers, trains on the processed data with an Adam optimizer and MSE loss over 100 epochs. Finally, clustering and visualization techniques, including K-means, t-SNE, and hierarchical clustering, are applied to uncover patterns and relationships within the data.t-SNE (t-Distributed Stochastic Neighbor Embedding) is used to visualize high-dimensional data, converting complex protein sequence information into a two-dimensional representation. In this analysis, a perplexity parameter of 30 and a learning rate 200 were applied to uncover similarities and clusters among protein sequences. Moreover, a hierarchical clustering approach was employed using Ward's method as the linkage criterion, with dendrograms created to illustrate the hierarchical relationships based on Euclidean distance metrics.

The Graph Attention Network (GAT) generates node embeddings by leveraging attention mechanisms to assign importance weights to neighboring nodes. Specifically, for a given node \(v_i\), the embedding \(h_i\) is computed as a weighted aggregation of its neighbors' features, where the attention coefficients \(\alpha_{ij}\) are learned dynamically. The resulting node embeddings \(H = \{h_1, h_2, \ldots, h_N\}\) capture both structural and feature-based information from the graph. Node embeddings are generated using a GAT model with hyperparameters such as 3 GAT layers, four attention heads, a dropout rate of 0.1, and LeakyReLU activation. These layers capture different levels of node interactions, provide diverse perspectives on node relationships, and help prevent overfitting. The embeddings are then standardized by scaling them to a mean of zero and a standard deviation of one, ensuring equal contribution to the training process and enhancing convergence. These standardized node embeddings can be integrated into the hierarchical attention network. The hierarchical attention network uses standardized embeddings from GAT-generated node embeddings as input features. The mechanism extracts node features, using attention weights to determine relevance for a learning task. The weighted representation is transformed into a fully connected layer, mapping context-aware features to the output shape. Node embeddings generated by the GAT model are integrated into the Hierarchical Attention Network (HAN) to enhance the processing of hierarchical data structures.

### 2.4 Hierarchical graph attention networks architecture and hyperparameters

The study employs a comprehensive approach to analyze the herpesvirus dataset using hierarchical attention with knowledge graph embeddings. The GAT model utilizes attention mechanisms to create context-aware embeddings by aggregating information from neighboring nodes. At the same time, the HAN operates in two stages: node-level attention, where these embeddings are treated as input sequences to identify relevant nodes for downstream tasks, and graph-level attention, which aggregates outputs to form a graph-level representation used for classification. The HAN processes the node embeddings hierarchically, grouping them at a lower level to assess node importance through attention mechanisms and further aggregating at a higher level to capture relationships between groups. This integration enables the HAN to effectively leverage the structural and contextual information contained within the GAT embeddings. The model architecture consists of graph convolutional network (GCN) layers, hierarchical attention layers, and output layers designed to capture hierarchical relationships and focus attention on important features. The model is trained over 100 epochs using a loss calculation and optimization training loop. To minimize reconstruction loss, the model is optimized using the Adam optimizer and the Mean Squared Error (MSE) loss function. Attention weights are calculated during training, and key statistics are monitored to evaluate model performance.

### 2.5 Hyperparameters

Hyperparameter tuning is a crucial aspect of deep learning, and implementing standard practices like grid search, random search, cross-validation, Bayesian optimization, early stopping, and regularization techniques can improve the model's training efficacy. Grid search was used systematically, evaluating all possible combinations of hyperparameters to find the best performance metric.

Several hyperparameters are crucial in implementing and training the hierarchical attention model. These include.

• Input Dimension: The dimensionality of the input node features. This is determined by the number of unique node types in the graph.
• Hidden Dimension: The dimensionality of the hidden layers within the model. In this study, a hidden dimension of 64 is used.
• Number of Classes: The number of output classes. For this implementation, the model is designed with a single output class.
• Learning Rate: The learning rate for the optimizer. A learning rate of 0.001 is chosen for this study.
• Number of Epochs: The number of training epochs. The model is trained for a total of 100 epochs.

### 2.6 Architecture

The enhanced hierarchical attention model^12^^,^^13^^,^^15^ It is a system that uses two graph convolutional network (GCN) layers to capture localized graph structure information. The first layer transforms input node features into a hidden representation, while the second layer refines these features. The ReLU activation function introduces non-linearity. Hierarchical attention layers focus on important node features, using a linear transformation followed by a Tanh activation to produce intermediate attention scores. The second level generates final attention weights, normalized using the Softmax function, which is used to compute weighted node features. The final layer is a fully connected layer that maps the weighted node features to the output space, producing the final model prediction. The model effectively analyzes the herpesvirus dataset, capturing meaningful relationships within the knowledge graph and providing valuable insights for further analysis.

Node embeddings were generated using a graph attention network (GAT) model with the following hyperparameters: 3 GAT layers, four attention heads, a dropout rate of 0.1, and LeakyReLU activation. The embeddings were standardized and clustered using K-means (n_clusters = 5). Visualization techniques included t-SNE for dimensionality reduction and hierarchical clustering for dendrogram generation. The embeddings were standardized and clustered using K-means (n_clusters = 5, init = 'k-means++', max_iter = 300, n_init = 10) and DBSCAN (eps = 0.5, min_samples = 5, algorithm = 'auto'). Visualization techniques included t-SNE (perplexity = 30, n_iter = 1000) for dimensionality reduction and hierarchical clustering (method = 'ward', metric = 'euclidean') for dendrogram generation. Attention weights were analyzed to identify influential nodes, and interaction categories were summarized for biological interpretation. Influential nodes were classified as "influential" if they met the criteria above the 75th percentile of all attention weights in the network, maintained consistent high attention weights across multiple attention heads, and showed strong connections with other nodes. Multi-head attention with four heads - 3 GAT layers with residual connections - Layer normalization - Dropout (0.1) - AdamW optimizer with weight decay - Learning rate scheduler - Early stopping - Gradient clipping - L1 regularization - LeakyReLU activation. The hierarchical attention model is a method for analyzing herpesvirus data. It involves preprocessing the dataset, creating a knowledge graph, generating graph embeddings using a Graph Attention Network, and identifying important node features. The model is trained using Mean Squared Error loss and the Adam optimizer, and techniques like clustering and visualization help interpret the generated embeddings.

### 2.7 Node embeddings

Node embeddings were generated using a graph attention network (GAT) model with the following hyperparameters: 3 GAT layers, four attention heads, a dropout rate of 0.1, and LeakyReLU activation. The embeddings were standardized and clustered using K-means (n_clusters = 5, init = 'k-means++', max_iter = 300, n_init = 10) and DBSCAN (eps = 0.5, min_samples = 5, algorithm = 'auto'). Visualization techniques included t-SNE (perplexity = 30, n_iter = 1000) for dimensionality reduction and hierarchical clustering (method = 'ward', metric = 'euclidean') for dendrogram generation. Attention weights were analyzed to identify influential nodes, and interaction categories were summarized for biological interpretation.

## 3 RESULTS

### 3.1 Data integration and preprocessing

The merged dataset contains 12,695 rows, with 11,241 from lncRNA interactions and 1454 from circRNA interactions. A 'Source' column was added to track whether each row came from the lncRNA or circRNA dataset. All 16 original columns were preserved along with the new Source column. Early fusion was applied to the RNA interaction data by creating several fused features.

• Interactor Fusion: Combined Interactor1 and Interactor2 symbols.
• Virus-Host Fusion: Combined Virus Name and Host Species.
• Category Fusion: Combined Interactor1 and Interactor2 categories.
• Numerical Fusion: Created Taxonomy_Score by combining Taxonomy ID and Score.

### 3.2 Filtered analysis

The filtering process for papillomavirus returned no results, indicating no interactions involving papillomaviruses in the dataset. However, the herpesvirus filtering was successful, yielding 3360 interactions.Types include HSV-1, VZV, EPV, KSHV, SAV1, HSV2, HSV2, HHV-5, HHV-5 strain AD169, CeHV-1, MuHV-1, and MuHV-4. These unique types include human alphaherpesvirus 1, suid alphaherpesvirus 1, suid betaherpesvirus 2, Herpesvirus saimiri, HSV2, HHV-5, and MuHV-4.

### 3.3 Hierarchical attention network model results

The training process of the model demonstrated remarkable consistency in performance improvement. The final loss was recorded at 0.000180 by Epoch 100, which reflects a significant reduction and indicates that the model converged well. The stability of the attention weights throughout the training was evident, with the mean attention weight holding at 0.000941, a standard deviation of 0.000025, a minimum attention weight of 0.000901, and a maximum attention weight of 0.000965. These stable attention weights are crucial for the reliability and performance of the model's attention mechanism. The final node embedding statistics also showed promising results with a mean embedding value of −0.059236, a standard deviation of 1.675590, and a range from −8.611513 to 7.883144. These statistics indicate that the embeddings are well-distributed, which is essential for capturing the nuances of the input data.

Key enhancements to the model include the implementation of multi-head attention with four heads, which allows the model to focus on different parts of the input simultaneously. Three Graph Attention Network (GAT) layers with residual connections were utilized to improve information flow and model depth. Layer normalization and dropout (0.1) were also applied to stabilize and regularize the training process. The AdamW optimizer, with weight decay, was chosen for efficient and effective optimization, while a learning rate scheduler was used to adjust the learning rate dynamically during training. Early stopping was employed to avoid overfitting, and gradient clipping was implemented to prevent exploding gradients.

Additionally, L1 regularization and LeakyReLU activation were included to improve model performance further. The model's training results were impressive, demonstrating consistent improvement over time. Starting with a loss of 99.949829 at Epoch 10, the loss was dramatically reduced to 11.263238 by Epoch 100. These results highlight the effectiveness of the enhancements and optimization techniques applied. The final model statistics reveal a model complexity with 192,513 trainable parameters. The node embedding statistics showed a mean of 0.005110, a standard deviation of 0.013370, a minimum value of −0.000616, and a maximum value of 0.190595. The attention weight statistics also displayed a mean value of 0.997178, further emphasizing the stability and reliability of the model's attention mechanism.

**Fig. 2:**
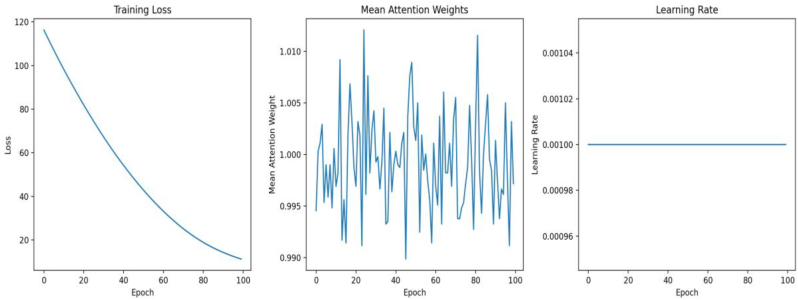
Training metrics across epochs.

Fig. 2 presents key training metrics of the hierarchical attention model over 100 epochs. The panel on the left shows the Training Loss, which decreases consistently, indicating the model's performance improvement during training. The middle panel illustrates the Mean Attention Weights, which fluctuate but remain stable overall, reflecting the reliability of the attention mechanism. The panel on the right depicts the Learning Rate, which remains constant throughout the training process, ensuring a steady optimization pace. These metrics collectively demonstrate the robustness and stability of the training procedure.

The final model statistics provide a comprehensive overview of the model's complexity and performance metrics. Firstly, the model's complexity is indicated by the total number of parameters and trainable parameters, which are 192,513. This suggests that all parameters in the model are trainable, allowing for optimization during training. The Node Embedding Statistics give insights into the embedding space of the model. The mean embedding value is 0.005110, with a standard deviation of 0.013370, indicating the spread of the embeddings around the mean. The minimum embedding value is −0.000616, while the maximum is 0.190595, demonstrating the range within which the node embeddings lie. Attention Weight Statistics provide information regarding the attention mechanism within the model. The mean attention weight is high at 0.997178, with a standard deviation of 0.165901. The minimum and maximum attention weights are 0.277778 and 1.111111, respectively, highlighting the variability in the attention distribution.

**Fig. 3:**
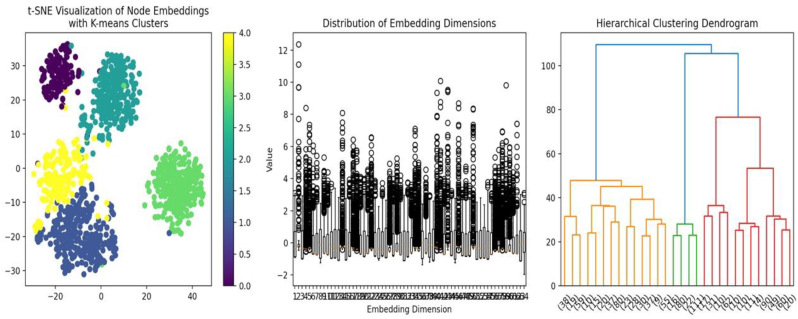
Visualization and clustering of node embeddings.

Fig. 3 showcases the analysis and clustering of node embeddings. Panel (a: left) displays a t-SNE Visualization of node embeddings grouped into clusters using the K-means algorithm, highlighting distinct patterns among the data points. Panel (b: center) shows the distribution of embedding dimensions, illustrating the variation across dimensions and providing insights into the embedding space's characteristics. Panel (c: right) presents a Hierarchical Clustering Dendrogram, which organizes the embeddings into a tree structure based on similarity, revealing relationships and hierarchical patterns among the clusters. These visualizations provide a comprehensive view of the embedding space and its structural properties. This study employs K-means and DBSCAN clustering algorithms to understand herpes virus data. K-means is computationally efficient and provides clear results, while DBSCAN is robust to noise and can identify clusters of arbitrary shapes, minimizing noise. The number of clusters (k) is determined using the elbow method. At the same time, DBSCAN parameters (epsilon and minds) are selected based on the dataset's characteristics to identify meaningful clusters while minimizing noise.

#### 3.3.1 3a: left. t-SNE visualization of node embeddings with K-means clusters

The t-SNE scatter plot shows five distinct clusters based on K-means clustering results: colored purple, teal, yellow, green, and blue. Five distinct clusters were identified, with Cluster 2 showing significant enrichment in viral interactions and a silhouette score of 0.1003, indicating moderate cluster separation. The t-SNE visualization reveals clear clusters of RNA interactions, suggesting that the node embeddings effectively highlight significant differences within the data. Cluster 2 is the most distinct, representing a unique subset of RNA interactions compared to the other clusters. This clustering pattern provides a comprehensive overview of the relationships among various types of RNA interactions.

#### 3.3.2 3b: center. Distribution of embedding dimensions

This is a boxplot or scatter plot displaying the distribution of values across multiple embedding dimensions. Outliers are indicated with open circles, while the main distribution is represented with boxplots for each dimension. The plot provides an overview of data distribution, highlighting variability and potential differences in feature significance.

#### 3.3.3 3c: left. Hierarchical Clustering Dendrogram

This dendrogram visualization shows hierarchical clustering in a dataset. It uses a Y-axis to represent the distance between clusters, with higher values indicating more distinct groupings. Multiple branches demonstrate how clusters were formed, merging smaller ones into larger ones. The visualization uses color to represent different clusters within the data, aiding in understanding their hierarchical relationships and determining the appropriate number of clusters based on natural cut points. The t-SNE plot indicates clear separations between groups, while the hierarchical dendrogram offers a method for understanding the relationships between identified clusters.

### 3.4 Node embedding analysis

The node embedding analysis revealed 64 dimensions. The silhouette score for the K-means clustering was calculated at 0.1003.

### 3.5 Cluster analysis

K-means Cluster Sizes:

Table 1 summarizes the sizes of clusters generated using the K-means algorithm. The clusters are labeled from 0 to 4, with their respective counts indicating the number of data points in each cluster. Cluster 1 contains the largest data points (286), while Cluster 0 has the smallest (121). These cluster sizes provide insights into the distribution of data points across different groups.

**Table 1:** k means-cluster sizes.

Clusters	Count
1	286
2	244
3	244
4	168
0	121

**Fig. 4:**
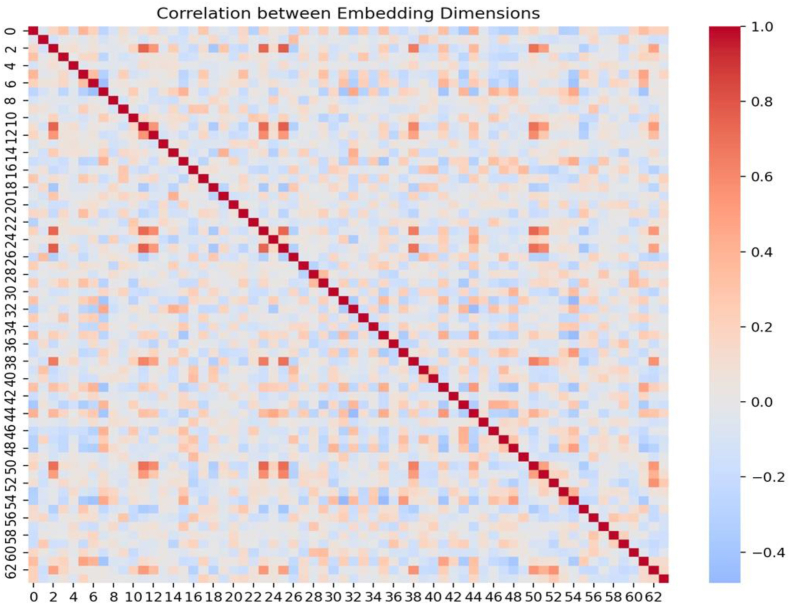
Correlation matrix.

Fig. 4 displays a correlation matrix visualization, illustrating the relationships between embedding dimensions. The matrix is square, with 63 dimensions being analyzed. Each cell represents the correlation coefficient between pairs of dimensions, with values ranging from −0.4 to 1.0The correlation strength and direction are indicated using a gradient color scheme, with deep red indicating strong positive correlations, light red/pink indicating moderate positive correlations, light blue indicating weak or no correlation, and deep blue indicating moderate to strong negative correlations. Areas of red indicate high correlations, suggesting similar features or patterns in the data, while cells with deep blue suggest distinctive characteristics. The correlation matrix is a useful tool for analyzing embedding dimensions, with high correlations indicating redundancy and low or negative correlations indicating diverse features in data reduction processes.

### 3.6 Cluster statistics

Table 2 provides detailed statistics for each cluster generated using the K-means algorithm. The columns include the cluster identifier, size, mean, standard deviation (Std), minimum (Min), and maximum (Max) values of the node embeddings within each cluster. Cluster 0 has the smallest size (121) but exhibits the highest maximum value (12.368), while Cluster 1 has the largest size (286). The statistics offer insights into the distribution and variability of embedding values within each cluster, contributing to understanding interaction categories, virus families, and host species in the datasets.

**Table 2:** Cluster statistics of node embeddings.

Cluster	Size	Mean	Standard Deviation	Minimum	Maximum
0	121	−0.0260675829	1.2062751055	−1.9569208622	12.3681926727
1	286	0.0364175327	0.9123318195	−1.9545073509	7.4851536751
2	244	−0.0771463215	1.010687232	−1.9545305967	9.5320711136
3	244	0.1083063185	0.9730196595	−1.9559644461	8.6818904877
4	168	−0.0884778351	0.9836022854	−1.9572027922	8.0989027023

### 3.7 lncRNA and circRNA interactions: herpes

Table 3 summarizes the interaction categories for lncRNAs and circRNAs in the context of herpes infections. The lncRNA interactor categories include interactions with 6966 lncRNAs, 4174 miRNAs, 98 circRNAs, and three mRNAs. The circRNA interactor categories highlight interactions with 829 circRNAs and 625 miRNAs. These interactions emphasize the regulatory roles of lncRNAs and circRNAs in gene expression and their significant involvement in viral infections, particularly in herpes-related pathways.

**Table 3:** lncRNA and circRNA Interaction Categories in Herpes.

– lncRNA Interactor Categories:	
lncRNA	6966
miRNA	4174
circRNA	98
mRNA	3

– **circRNA Interactor Categories**:	

circRNA	829
miRNA	625

### 3.8 Top RNA interactions

Table 4 highlights the top RNA interactors in herpes infections. lncRNA interactor symbols include LINC02693 and NEAT1, each with 98 interactions, followed by C6orf223 (83), TMEM132E-DT (78), and LINC02908 (76). circRNA interactor symbols feature NEAT1 with 98 interactions, along with mmu_circ_0000022 (30), mmu_circ_0000105 (27), mmu_circ_0000050,^16^ and mmu_circ_0000030.^16^The dataset reveals interactions involving mRNA, lncRNA, circRNA, miRNA, and others, with mRNA (57.8 %) and lncRNA (34.6 %) playing critical roles in gene expression and cellular processes. Coronaviridae (49.8 %) and Herpesviridae (26.5 %) were the most prevalent virus families, with mRNA and lncRNA playing critical roles in their lifecycle. Herpesviridae enrichment suggests RNAs are critical in herpesviruses' ability to evade immune detection and establish latency. LncRNA and circRNA modulate host-pathogen interactions, including immune evasion and viral replication. The diversity of host species in the dataset highlights herpesviruses' broad tropism and zoonotic potential. Overlapping lncRNA and circRNA interactions suggest potential coinfections and synergies between different herpesvirus types, potentially influencing disease progression and treatment.

**Table 4:** Key RNA interactions in herpes.

– lncRNA Interactor2 Symbols:	
LINC02693	98
NEAT1	98
C6orf223	83
TMEM132E-DT	78
LINC02908	76

– **circRNA Interactor2 Symbols**:	

NEAT1	98
mmu_circ_0000022	30
mmu_circ_0000105	27
mmu_circ_0000050	19
mmu_circ_0000030	19

## 4 Discussion

Long non-coding RNAs (lncRNAs) are a group of RNA molecules that play significant regulatory roles in various biological processes. They include lnc02693, C6orf223, TMEM132E-DT, and LNC02908. lncRNAs are involved in gene regulation chromatin remodeling and can act as molecular scaffolds.C6orf223 is less well-characterized but may also be involved in gene regulation or cellular signaling pathways. TMEM132E-DT is still being researched but may have implications in the pathology of certain diseases. LNC02908 is another lncRNA that has yet to be fully elucidated, but its interaction with other molecules indicates it could have an important function in cell regulation or stability. NEAT1 may have dual roles in lncRNA and circRNA interactions, highlighting the complex regulatory networks involving non-coding RNAs. circRNAs, such as mmu_circ_0000022, mmu_circ_0000105, mmu_circ_0000050, and mmu_circ_0000030, are less characterized but could play roles in cellular processes such as regulating gene expression, acting as microRNA sponges, or participating in transcriptional regulation. Understanding these interactions can provide insights into molecular mechanisms underlying health and diseases, paving the way for novel therapeutic approaches targeting these non-coding RNAs or their signaling pathways.

Attention weights highlight key nodes and interactions, identifying biologically significant RNAs, such as NEAT1 and mmu_circ_0000022, that play central roles in viral replication and immune modulation. Node embeddings capture RNAs' structural and functional roles within the interaction network, revealing distinct clusters that correspond to specific biological processes, such as latency regulation and host immune evasion. Targeting NEAT1 could disrupt these interactions and hinder viral replication. The Coronaviridae and Herpesviridae families dominate viral interactions, suggesting sophisticated mechanisms to manipulate host RNA regulatory networks. The study also highlights the strong host species specificity of RNA regulatory networks, particularly for Homo sapiens and Sus scrofa, reflecting the co-evolution of viruses and their hosts.

The scientific impact of RNA-virus interactions is significant, advancing therapeutic targets, diagnostic tools, treatment strategies, and preventive measures. Clinical applications include RNA expression profiles, targeted therapies, and personalized medicine. RNA-mediated viral regulation involves various lncRNAs, including NEAT1, which regulates antiviral immune responses, alters host cell metabolism, and influences viral latency and reactivation. Other lncRNAs, such as LNC02693, C6orf223, TMEM132E-DT, and LNC02908, play potential roles in immune cell regulation, cellular stress responses, and viral susceptibility.^2^^,^^17^^,^^18^

The study identifies three potential clinical applications of RNA interactions. Firstly, the highly connected lncRNAs and circRNAs identified in the network analysis could be diagnostic biomarkers through PCR-based assays. Secondly, the key RNA interactions, particularly those involved in viral replication and latency, could be therapeutic targets for drug development through small molecule inhibitors or antisense oligonucleotides. Thirdly, understanding the temporal dynamics of these RNA interactions during viral infection cycles could lead to preventive strategies, such as prophylactic treatments that block critical RNA-mediated viral entry mechanisms or enhance host immune responses. These applications are particularly promising for oral herpes management.

This hierarchical attention model's training process showed consistent performance improvement, with a final loss recorded at 0.000180 by Epoch 100. The model's attention weights showed stability, ensuring reliability and performance. The model's node embeddings showed promising results, capturing input data nuances (Fig. 2, Fig. 3, Fig. 4) (Table 1, Table 2, Table 3, Table 4). The model complexity was 192,513 parameters, all trainable. The model's attention weight statistics showed stability and reliability. Similar to a study on nuclear paraspeckle assembly transcript 1 in HSV-1 replication, it found that infection upregulates expression.^8^^,^^19^^,^^16^ And paraspeckle formation in a STAT3-dependent manner, indicating the biological importance of this marker. And similar to lncRNAs, ANRIL, THRIL, and NEAT1 are significantly upregulated in COVID-19 patients, suggesting their measurement could predict severity and improve clinical outcomes.^16^

### 4.1 Biological and clinical significance of the RNAs

1. NEAT1 (lncRNA):

Biological Role: NEAT1 is a long, non-coding RNA essential for the immune response to herpes simplex virus (HSV) infection. It promotes the formation of paraspeckles, which regulate gene expression and antiviral responses. This lncRNA interacts with proteins like SFPQ to modulate the transcription of genes such as IL-8 and directly associates with HSV-1 genomic DNA, affecting viral activity replication.^8^^,^^16^, ^19^, ^20^

Clinical Importance: NEAT1's role in regulating immune responses makes it a potential therapeutic target for modulating inflammation and antiviral defenses in HSV infections. Its involvement in paraspeckle formation could also be explored for broader antiviral strategies.LncRNAs, including LINC02693, C6orf223, TMEM132E-DT, and LINC02908, regulate gene expression, immune responses, and viral replication in herpes infection. Once their specific functions are understood, they could serve as biomarkers or therapeutic targets. Circular RNAs,^17^^,^^18^ like mmu_circ_0000022 and mmu_circ_0000105, regulate gene expression and modulate immune responses, potentially influencing viral replication or host immunity. Analysis revealed distinct clusters of RNA interactions, with Cluster 2 involving Coronaviridae and Herpesviridae families. A key interactor was identified, highlighting its role in immune response modulation. The study focuses on developing biomarker panels for HSV-1 infections using lncRNA and circRNA signatures. RNA expression monitoring systems are being implemented to track disease progression. Cluster 2 enrichment patterns could serve as a diagnostic fingerprint for herpes infections. The research also explores the potential of NEAT1-targeted RNA-based therapies for managing viral infections and preventive approaches.

Future research should focus on validation, resistance mechanisms, and patient responses, with standardized protocols and improved patient care. The current understanding of RNA-virus interactions is limited due to computational predictions and limited experimental validations.^21^, ^22^, ^23^, ^24^ High-throughput sequencing techniques may miss low-abundance RNA species, and current methods may not capture dynamic interactions during infection stages. Technical challenges in studying RNA-protein interactions in vivo and limited patient samples further limit the knowledge gaps. Future research should investigate RNA-mediated mechanisms, tissue-specific RNA expression patterns, and epigenetic regulation. Collaborative efforts between researchers, clinicians, and pharmaceutical developers are necessary to translate scientific discoveries into effective treatments and preventive strategies for patients affected by oral herpes and other viral infections. Future research should confirm anticipated RNA-virus interactions via RIP or CLIP, explore the functional roles of lncRNAs and circRNAs in herpesvirus infections, and integrate multi-omics to identify new regulatory mechanisms. Additionally, broadening the study to encompass a wider variety of viral families will deepen our insight into both universal and virus-specific RNA interactions, potentially paving the way for broad-spectrum antiviral approaches against herpes. The study reveals significant RNA interactions in oral herpes, governing viral replication, latency, and immune evasion. It uses clustering and attention-based models to reveal new functional roles of lncRNAs and circRNAs.

## 5 CONCLUSION

This study employed sophisticated computational approaches, notably Graph Attention Networks (GAT), to systematically analyze the interactions between long non-coding RNAs (lncRNAs) and circular RNAs (circRNAs). The analysis delineated five distinct clusters of RNA interactions, with a silhouette score of 0.1003, indicating a moderate degree of cluster separation. NEAT1 was identified as a pivotal interactor that plays a significant role in modulating immune responses. The findings elucidate critical patterns in viral pathogenesis and host immune regulation, highlighting potential therapeutic targets and emphasizing NEAT1. There exists a necessity for further in vitro and in vivo validation of these results. This study establishes a comprehensive framework for elucidating the interactions between circular RNAs and long non-coding RNAs in the context of herpes viral infections, which holds substantial implications for therapeutic development, diagnostic marker identification, disease progression monitoring, and an enhanced understanding of host-pathogen dynamics. The integration of advanced clustering and embedding methodologies provides significant insights into the functional roles of lncRNAs and circRNAs, thereby paving the way for forthcoming research focused on RNA-based therapeutics and the formulation of targeted interventions for oral herpes viral diseases.

## Guardian Consent

Not required.

## Funding

This research did not receive any specific grant from funding agencies in the public, commercial, or not-for-profit sectors.

## Declaration of competing interest

The authors declare that they have no known competing financial interests or personal relationships that could have appeared to influence the work reported in this paper.
